# PCR Primers to Study the Diversity of Expressed Fungal Genes Encoding Lignocellulolytic Enzymes in Soils Using High-Throughput Sequencing

**DOI:** 10.1371/journal.pone.0116264

**Published:** 2014-12-29

**Authors:** Florian Barbi, Claudia Bragalini, Laurent Vallon, Elsa Prudent, Audrey Dubost, Laurence Fraissinet-Tachet, Roland Marmeisse, Patricia Luis

**Affiliations:** 1 Ecologie Microbienne, UMR CNRS 5557, USC INRA 1364, Université de Lyon, Université Lyon 1, Villeurbanne, France; 2 Department of Life Sciences and Systems Biology, University of Turin, Turin, Italy; Friedrich Schiller University, Germany

## Abstract

Plant biomass degradation in soil is one of the key steps of carbon cycling in terrestrial ecosystems. Fungal saprotrophic communities play an essential role in this process by producing hydrolytic enzymes active on the main components of plant organic matter. Open questions in this field regard the diversity of the species involved, the major biochemical pathways implicated and how these are affected by external factors such as litter quality or climate changes. This can be tackled by environmental genomic approaches involving the systematic sequencing of key enzyme-coding gene families using soil-extracted RNA as material. Such an approach necessitates the design and evaluation of gene family-specific PCR primers producing sequence fragments compatible with high-throughput sequencing approaches. In the present study, we developed and evaluated PCR primers for the specific amplification of fungal CAZy Glycoside Hydrolase gene families GH5 (subfamily 5) and GH11 encoding endo-β-1,4-glucanases and endo-β-1,4-xylanases respectively as well as Basidiomycota class II peroxidases, corresponding to the CAZy Auxiliary Activity family 2 (AA2), active on lignin. These primers were experimentally validated using DNA extracted from a wide range of Ascomycota and Basidiomycota species including 27 with sequenced genomes. Along with the published primers for Glycoside Hydrolase GH7 encoding enzymes active on cellulose, the newly design primers were shown to be compatible with the Illumina MiSeq sequencing technology. Sequences obtained from RNA extracted from beech or spruce forest soils showed a high diversity and were uniformly distributed in gene trees featuring the global diversity of these gene families. This high-throughput sequencing approach using several degenerate primers constitutes a robust method, which allows the simultaneous characterization of the diversity of different fungal transcripts involved in plant organic matter degradation and may lead to the discovery of complex patterns in gene expression of soil fungal communities.

## Introduction

In forest ecosystems, up to two thirds of the organic carbon (C_org_) are stored in soils and a large part is localized in plant litters [1]. As litter input can exceed 3.5 t ha^−1^ yr^−1^, it represents, along with root exudates, the main source of soil organic matter (OM) and its degradation by soil organisms is essential for carbon cycling [2], [3]. Plant litter decomposition is largely controlled by soil fauna and microorganisms (bacteria, fungi…). In terrestrial ecosystems, the above- and belowground plant litter constitutes the main source of energy and matter for the soil heterotrophic microflora [4]. Soil microorganisms have developed strategies to drive plant-residue mineralization by producing a large number of extracellular enzymes [5].

Cellulose, hemicelluloses and lignin are the three most abundant biopolymers in plant litter and in soil OM derived from its decomposition [6]. Lignin, a polymer highly recalcitrant to enzymatic degradation, restricts microbial and enzyme access to cellulose and other labile carbon compounds that it protects [7]. Saprotrophic fungi are considered to be the most efficient decomposers of these biopolymers due to their important lignocellulolytic potential and wide diversity in soils [8], [9], [10]. Indeed, the complete breakdown of lignin can only be achieved by saprotrophic basidiomycetes whereas a larger number of soil fungi are able to perform the degradation of polysaccharides such as cellulose and hemicelluloses [10]. Schneider et al. [11] reported, using a metaproteomic approach, that all hydrolytic enzymes they could identify from a beech litter extract were likely of fungal origin.

Forest soil fungal communities and their associated functions can be affected by several environmental factors including seasonal climatic cycles, stand age, tree species and therefore the quality of litter they produce [12]–[16]. Within a given climate, litter quality is the overriding factor controlling decomposition rate [17]. Activities expressed by single fungal species are difficult to access *in situ* with conventional approaches. Methods commonly applied in soil surveys such as enzymatic activities or respiration rate measurements do not indicate which fungi are responsible for these processes [18]. Determination of the functions of fungal species, which typically requires their isolation in pure culture and the study of their effects on defined substrates, has well-documented limitations [19]–[21]. The use of degenerate primer sets for fungal functional gene amplification and their utilization on nucleic acids extracted from soil samples provides cultivation-independent tools for assessing the genetic diversity and activity of lignocellulolytic degrading guild within fungal communities [22]–[27]. Relevant and unexpected information was revealed by the utilization of these approaches such as the potential role of Ascomycota species and certain ectomycorrhizal fungi in lignin degradation in soils [18], [26]. Thus far, two main fungal enzyme-coding gene families have been targeted in environmental studies; the laccases (EC 1.10.3.2) of the CAZy Auxiliary Activity (AA) family 1 whose role in lignin breakdown in the field has always been controversial [28] and the CAZy Glycoside Hydrolase (GH) family 7 encoding essentially either endo-β-1,4-glucanases (E.C. 3.2.1.4) or cellobiohydrolases (E.C. 3.2.1.176), both active on cellulose [22], [25], [29]–[33].

As the three major components of plant litter are cellulose, hemicelluloses and lignin, simultaneous study of fungal communities participating to their breakdown would require PCR amplification and sequencing of several key enzyme-coding gene families known to be active on these polymers. In the present study we developed, evaluated and tested on soil-extracted RNA samples PCR primers targeting functional genes active on these three plant cell wall constituents. For cellulose, in addition to the well-documented GH7 family, we developed primers for the subfamily 5 of the GH5 family (GH5-5). As the GH5 family encompasses enzymes active on a wide range of substrates (i.e. cellulose, mannane, chitosane…) which are distributed in different subfamilies (GH5-1, GH5-2, GH5-3…), we specifically targeted the subfamily GH5-5 known to comprise only endo-β-1,4-glucanases (E.C. 3.2.1.4) [34]–[35]. The members of this GH5-5 subfamily are widely distributed among fungi [35] and are often highly expressed in presence of cellulose in saprotrophic species [36]–[38]. For hemicellulose, we targeted the fungal GH11 encoding only endoxylanases and almost exclusively endo-β-1,4-xylanases (EC 3.2.1.8) [34]. Xylan constitutes the major component of hemicelluloses and its proportion is generally higher in broad-leaved trees compared to coniferous ones [39]. For lignin, we focused on the Basidiomycota class II peroxidases corresponding to CAZy Auxiliary Activity family 2 (AA2) which comprises Manganese (MnP; EC 1.11.1.13), lignin (LiP; EC 1.11.1.14), versatile (VP; EC 1.11.1.16) and generic (GP; 1.11.1.7) peroxidases. These peroxidases, which can display oxidizing activities towards aromatic compounds such as lignin, have a complex evolutionary history [40] and have been essentially studied in wood rot fungi although they have also been reported in a wide range of soil-borne Basidiomycota including symbiotic ectomycorrhizal species [24], [26]–[27], [41].

The present study includes different aspects. We initially designed, *in silico*, PCR primers based on sequence alignments and then tested them on a wide range of DNA samples corresponding to fungi belonging to different classes of Asco- and Basidiomycota, including species with sequenced genomes for which the presence/absence of the studied gene families had been established. We then evaluated the suitability of several of the resulting PCR products for high-throughput sequencing using the Illumina MiSeq approach. Amplifications were performed on cDNAs synthesized from soil-extracted RNA samples obtained from two forest stands differing with respect to their dominant tree species (spruce versus beech). Working on environmental RNA, instead of DNA, gives access to the active fraction of the soil community. Furthermore, in the case of eukaryotes, it also circumvents the problem of introns, which can either disrupt PCR primer binding sites or create large size variations between PCR products.

## Materials and Methods

Concerning fieldwork permits, soil samples were collected in the framework of collaborative projects including Jacques Ranger and Arnaud Legout from the “Institut National de la Recherche Agronomique” (INRA) of Nancy (France) who are in charge of the Breuil-Chenue site.

### Primer design and analysis of their efficiency on fungal DNA

New degenerate primers (Table 1) were designed, according to the process described by Kellner et al. [18], to specifically amplify fungal genes encoding endo-β-1,4-glucanases (EC 3.2.1.4) of the GH5 subfamily 5, endo-β-1,4-xylanases (EC 3.2.1.8) of the GH11 family, and Basidiomycota-class II peroxidases (EC 1.11.1.13 (MnP), EC 1.11.1.14 (LiP), EC 1.11.1.16 (VP) and EC 1.11.1.7 (GP)) of the AA2 family. These primer pairs were developed on the basis of reference protein sequences retrieved from the curated CAZy [34] and GenBank [42] databases. The CAZy reference sequences (S1 Table) were compared against the NCBI database using the standard protein-protein BLAST (blastp) and the distance tree option implemented in the NCBI result page was used to display the phylogenetic relationship of each protein among the different fungal groups. Several representatives of each clade were selected to generate multiple alignments. Degenerate primer pairs were then developed for conserved protein regions of each enzyme family or subfamily (S1 Table) to amplify either both Ascomycota or Basidiomycota (GH5-5 and GH11) or only Basidiomycota (AA2).

**Table 1:** Degenerate primers used in this study.

Gene family-Subfamily^1^	Primer namesand sequences (5′-3′)	PCR fragmentlength (bp)	Targetedfungal groups	Reference
GH7	fungcbhI-F: ACC AAY TGC TAY ACI RGY AA; fungcbhI-R: GCY TCC CAI ATR TCC ATC	515	Basidiomycota;Ascomycota	[23]
GH5-5	fungGH5-5-F: GAR ATG CAY CAR TAC CTY GA; fungGH5-5-R: CA NGG ICC RGC RGC CCA CCA	248	Basidiomycota;Ascomycota	This study
GH11	fungGH11-F: GGV AAG GGI TGG AAY CCN GG; fungGH11-R: TG KCG RAC IGA CCA RTA YTG	281	Basidiomycota;Ascomycota	This study
AA2	basidioAA2-F: GGY GGI GGI GCB GAY GGY TC; basidioAA2-R: GG RGT IGA GTC RAA NGG	398	Basidiomycota	This study

1 according to the CAZy database (http://www.cazy.org/; [34]). The GH7 family encodes essentially either fungal endo-β-1,4-glucanases (E.C. 3.2.1.4) or cellobiohydrolases (E.C. 3.2.1.176), both active on cellulose; the GH5-5 subfamily only encodes fungal endo-β-1,4-glucanases (E.C. 3.2.1.4); the GH11 family encodes only endoxylanases and almost exclusively fungal endo-β-1,4-xylanases (EC 3.2.1.8); The AA2 family comprises Manganese (MnP; EC 1.11.1.13), lignin (LiP; EC 1.11.1.14), versatile (VP; EC 1.11.1.16) and generic peroxidases (GP; 1.11.1.7).

The efficiency of each primer pair was tested on DNA extracted from 72 different fungal species, belonging to either the Basidiomycota or Ascomycota (S2 Table). Complete genomes were available for 27 of these fungal species at the JGI genome portal MycoCosm (http://genome.jgi-psf.org/programs/fungi/index.jsf; [43]) or at the Broad Institute website (https://www.broadinstitute.org). Fungal genomic DNA extraction was performed from mycelia or fresh fruit bodies as previously described [44]. For PCR amplifications, 60 ng of fungal DNA were added to a 25 µl reaction mixture containing 2.5 µl of 10X polymerase buffer (Invitrogen), 0.75 µl of MgCl_2_ (50 mM), 2.5 µl of dNTPs (2 mM each), 1 µl of 5 mg.ml^−1^ bovine serum albumin, 0.5 µl of each primer (20 µM) and 0.1 U of polymerase mix (1∶24 (U∶U) of Biorad iProof High-Fidelity DNA Polymerase : Invitrogen *Taq* DNA polymerase). Cycling conditions, performed on a Peltier Thermal Cycler 100 (MJ Research), were 94°C for 3 min; 45 cycles of 94°C for 45 s, 50°C for 45 s, 72°C for 45 s, followed by 72°C for 10 min. Control reactions without nucleic acid were always run in parallel. PCR products were extracted from agarose gel using the QIAquick Gel Extraction Kit (Qiagen), ligated to the plasmid pCR4-TOPO (TOPO TA Cloning kit for sequencing, Invitrogen) and introduced into chemically competent TOP 10 *E. coli* cells. After plasmid extraction, using the NucleoSpin Plasmid kit (Macherey Nagel), inserts were sequenced by Biofidal (Lyon, France) using M13 forward and reverse primers and the Sanger technology.

### Study site and soil sampling

The experimental site of Breuil-Chenue forest is located in the Morvan mountains, Burgundy, France (47°18′10″N, 4°4′44″E). The elevation is 640 m, mean annual rainfall is 1280 mm and mean annual temperature is 9°C. The parent rock is granite and the soil is an alocrisol with pH between 4.0 and 4.5 [45]. The site is an environmental research observatory set up in 1976 in order to study the effects of tree species substitution on the biochemical and biological functioning of the soil ecosystem. The original forest, composed of several broadleaved tree species was clear-cut and replaced by several mono-specific stands of either coniferous or broadleaved tree species. Soil samples were collected from beech (*Fagus sylvatica*) and spruce (*Picea abies*) stands (ca 1000-m^2^ plots with soil pH of 3.9 and mull humus layers) along a systematic sampling grid [46]. For each stand, 14 soil cores of 750 cm^3^ (8 cm in diameter, 15 cm in depth) were sampled in one plot in July 2007 and 14 soil cores were taken in three independent plots in July and October 2010. After removing the surface litter, the organic matter-rich horizons (depth±0–7 cm) of each soil core were homogenized separately and sieved (2 mm mesh size) to eliminate small debris and root fragments. For each sampling date, a single composite sample per plot was prepared by pooling 100 mL of each core. Subsamples of the composite samples were frozen and kept at −70°C.

### Soil RNA extraction, reverse transcription and PCR amplification

Total RNA was extracted as described in Damon et al. [46] from 78 to 104 g of soil for each composite sample. Three to four series of 40 extractions (0.65 g of soil each) were performed in parallel for each composite samples. RNA extracts obtained from soil samples collected in July and October 2010 were pooled by forest stand before reverse transcription. Samples were designated as BB2007 for Breuil Beech 2007, BS2007 for Breuil Spruce 2007, BB2010 for Breuil Beech 2010 and BS2010 for Breuil Spruce 2010. Double stranded cDNA was synthesized from 2 µg of total soil RNA using the Mint-2 cDNA synthesis kit (Evrogen). The optimal number of PCR cycles to maintain a balance between maintaining transcript representation and reducing nonspecific background amplification during the cDNA production was found to be 27 cycles. The resulting cDNAs were used as template to specifically amplify expressed fungal genes encoding endo-β-1,4-glucanases (GH5-5 subfamily), endo-β-1,4-xylanases (GH11 family), and Basidiomycota-class II peroxidases (AA2 family) by using primers developed in this study and cellulases (GH7 family) by using the degenerate primers designed by Edwards et al. [23]. All PCR amplifications were performed in quintuplet in 25 µl reaction mixtures containing 1 µl of cDNA, 2.5 µl of 10X polymerase buffer with 25 mM MgCl_2_ (Invitrogen), 2.5 µl of dNTPs (2 mM each), 0.5 µl of each primer (20 µM) and 0.1 µl polymerase mix (1∶24 (U:U) of Biorad iProof High-Fidelity DNA Polymerase : Invitrogen *Taq* DNA polymerase). Amplification conditions were 3 min at 94°C; either 35 or 45 cycles of 45 s at 94°C, 45 s at either 48°C for the GH7 primer set or 50°C for all other primers, 2 min at 72°C, followed by 10 min at 72°C. Control reactions with non-reverse transcribed mRNA and without nucleic acid were run in parallel.

### Amplicon sequencing by Sanger and Illumina MiSeq approaches

Amplicons from samples BB2007 and BS2007 were sequenced using a Sanger approach. PCR products were extracted from agarose gel and cloned in *E. coli* as described above. A total of 48 bacterial clones were randomly selected for each gene family and the inserts sequenced by AGOWA (Germany) using both M13 forward and reverse primers. PCR products obtained from samples BB2010 and BS2010 were subjected to an Illumina MiSeq sequencing. Amplicons from the five independent PCR reactions were pooled and directly purified using the Qiagen QIAquick PCR purification Kit (GH11 and GH7 families) or separated by electrophoresis and gel purified (AA2 family). DNA quantity was measured using a Qubit 2.0 fluorometer (Invitrogen) and the Qubit dsDNA HS assay kit (Invitrogen). For each sample (BB2010 and BS2010) an equimolar mix of the different PCR products was made and a paired-end sequencing was carried out on an Illumina MiSeq sequencer by FASTERIS (Switzerland) using a 2×250 bp sequencing kit as available at that time. GH5-5 amplicons could not be included in this MiSeq analysis as their sizes (<250 bp) were too divergent from the ones of the class II peroxidases (∼400 bp) and GH7 (∼515 bp).

### Sequence analysis

All Sanger sequences obtained from fungal DNA or soil cDNA were manually edited, corrected, and deposited at the EMBL European Nucleotide Archive (http://www.ebi.ac.uk/ena/) under accession numbers HG799539-HG799611 and FR875180-FR875286. Concerning the Miseq data (deposited at EMBL-ENA under the project number PRJEB7363 with fastq-files accession nos. ERR636005-ERR636006), paired-end reads were assembled using PandaSeq v.2.5 [47] and all sequences containing “N”s were filtered out. Assembled paired-end reads were then analyzed using Mothur v.1.33 [48]. As GH11 and AA2 amplicons were smaller than 400 bp, forward and reverse reads were confidently assembled and the resulting merged sequences were trimmed according to both primer sequences using Mothur. As the GH7 PCR products were of about 500 bp in length, forward and reverse reads could not be assembled and we limited our analysis to the first 210 nucleotides of the reads bordered by the GH7 forward primer. To ensure high quality data for analysis, assembled pair-reads containing homopolymers longer than 7 bp and more than two mismatches in any primer sequence were removed. Chimeric sequences were detected using the UCHIME algorithm [49] and removed from the datasets. Sequences were then clustered at a cutoff value of 95% sequence identity for the GH7 & GH11 families and 93% for the AA2 one. For each sequence cluster, the most abundant sequence was chosen as its representative.

The subfamily assignation of the partial GH5 sequences was performed by Dr. Bernard Henrissat (CNRS, Marseille) using Hidden Markov models specific for each of the GH5 subfamilies and that are used for the daily updates of the GH5 subfamily information in the CAZy database. The subfamily assignation of the partial AA2 was performed as described in Kellner et al. [27] by recording the presence or absence of specific amino acids in the protein sequences. All MnP sequences contain an aspartic acid residue, corresponding to the Asp-175 of the *Phanerochaete chrysosporium* MnP1 (AAA33744), which is crucial for Mn^2+^ oxidation. All LiP sequences display a tryptophan residue, equivalent to the Trp-171 of the *P. chrysosporium* LiPH8 (AAA53109), which is responsible for oxidation of phenolic compounds. All VP sequences possess these two amino acids, while GP sequences lack them both.

Diversity indices and richness estimators were calculated using EstimateS v.9.1.0. (http://viceroy.eeb.uconn.edu/estimates/) on subsamples containing the same number of sequences per sample (i.e. the sequence number obtained for the BB2010 sample) to eliminate the effect of sequencing effort.

Phylogenetic analyses were performed on deduced amino acid sequences, which were aligned using MUSCLE [50] to all homologous sequences retrieved from GenBank (http://www.ncbi.nlm.nih.gov/; [42]), CAZy (http://www.cazy.org/; [34]), PeroxiBase (http://peroxibase.toulouse.inra.fr/; [51]) databases and from the published fungal genome sequences available at the JGI and Broad Institute. Maximum-likelihood (ML) trees were generated with PhyML v.3.0 [52] using the WAG substitution model [53] as implemented in SeaView v.4 [54]. Robustness of the tree topology was tested by bootstrap analysis (1000 replicates). As there were too many homologous protein sequences in databases (i.e. more than 500 different sequences for the AA2 and GH7 families), the ML trees illustrated in the manuscript include ∼100 known protein sequences representative of the phylogenetic diversity of each gene family, including the ones generated in the present study. Moreover, all environmental sequence clusters, except singletons identified in only one of the two soil samples, were included in these phylogenetic trees drawn in FigTree v.1.4 (http://tree.bio.ed.ac.uk/software/figtree/).

## Results

### Primer specificity and efficiency on fungal DNA and soil cDNA

*In silico*-designed primers for fungal genes belonging to families GH5 (subfamily 5), GH11 and AA2 were first tested on DNA extracted from known fungal species belonging to either the Ascomycota or the Basidiomycota (S2 Table). Depending on the gene family or subfamily, PCR products of the expected size (i.e. ∼250 bp for GH5-5, ∼300 bp for GH11 and ∼400 bp for AA2 family) were obtained for 17 to 47 of the 72 fungal species tested (S2 Table). The sequencing of two cloned PCR products per gene for 28 of these 72 fungal species always gave sequences belonging to the expected gene family or subfamily (GH5-5, GH11 or AA2), thus demonstrating the specificity of the PCR primers. Furthermore, in 22% of the cases the two homologous sequences were different, as expected for genes often occurring as gene families in fungal genomes. All GH5 partial sequences obtained were assigned to the GH5-5 subfamily encoding only endo-β-1,4-glucanases. Concerning the AA2 family, all subfamilies could be amplified. Indeed, among the 13 different AA2 sequences obtained, 8 belonged to the MnP subfamily, 1 to the LiP subfamily, 2 to the VP family and 2 to the GP one (S2 Table).

As anticipated, both GH5-5 and GH11 primers, which were designed using both Ascomycota and Basidiomycota gene sequences, indifferently amplified genes from DNA extracted from either Ascomycota or Basidiomycota species distributed in different families, orders and classes of these taxonomic groups. On the contrary, AA2 primers, designed using sequences exclusively from Basidiomycota amplified sequences only from DNA extracted from species belonging to this taxon (S2 Table).

**Figure 1:**
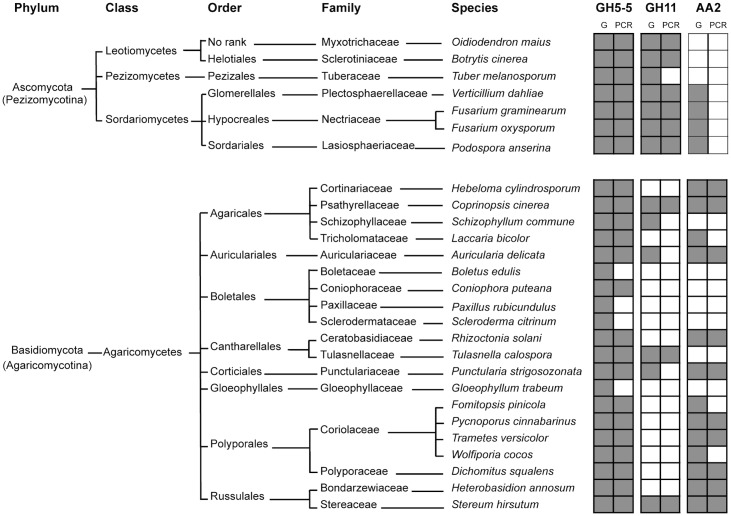
Primer specificity. Each pair of degenerate primers was tested on DNA extracted from 27 sequenced fungal species. Presence or absence of the GH5 (subfamily 5), GH11 and AA2 gene families in the published genomes (G) are indicated by gray and white boxes, respectively. Similarly, positive or negative PCR amplifications (PCR) are materialized by grey and white boxes respectively.

The effectiveness of the degenerate primers could also be evaluated from the amplification results obtained using DNA extracted from 27 species whose genome sequences, and therefore the presence or absence of the studied gene families, were known. Depending upon the gene family, positive amplifications were ranged from for 69% (9 of the 13 fungal species with predicted GH11 genes in their genomes) to 85% (23 of the 27 fungal species with predicted GH5 genes in their genomes) of the expected cases (Fig. 1). Positive amplifications were never observed when the genes were known to be absent. In cases of apparent amplification failure, examination of the genomic copie(s) of the corresponding gene families indicated that absence of amplification resulted essentially from highly divergent sequences at the PCR primer binding site(s) and to a lesser extent from the presence of introns within the primer region. Furthermore, for the same reasons, not all predicted copies in genomes are amplifiable with the designed primer sets. Concerning fungal species with predicted GH5-5 genes in their genomes, 33 to 100% of the genomic copies are amplifiable depending on the species (S2 Table). Regarding the GH11 genes, for most species, 33 to 50% of the predicted genomic copies are amplifiable. Based on the total number of predicted Class-II peroxidase sequences within the 21 Basidiomycota-genomes, our AA2 primers preferentially target MnP and LiP subfamilies and to a lesser extend GP and VP subfamilies as they potentially amplify 79% of LiP, 49% of MnP and only 12% of GP or VP genomic copies (S2 Table).

As the total RNA extracted from soil samples contains mRNA expressed by all soil organisms and not only by fungi, the specificity and efficiency of the designed degenerate primers (for the GH5-5 subfamily, GH11 and AA2 families) were also evaluated on soil cDNA by the sequencing of cloned PCR products. This analysis was carried out on the cDNA obtained from soil samples collected in 2007 in a beech (sample BB2007) and a spruce (sample BS2007) forest stand. For each cDNA sample, a total of 48 sequences were analyzed for each gene family or subfamily (i.e. GH5-5, GH7, GH11 and AA2). Among these sequences, between 8% and 29% of them were of bad quality and the presence of chimeric sequences was detected for the AA2 gene fragments from the beech forest soil (S3 Table). Analysis of the remaining high-quality sequences showed that all of them were homologous (with percentages of similar amino acid positions above 70%) to fungal lignocellulolytic enzyme sequences of the corresponding families/subfamilies already deposited in public databases (S3 Table). All GH5 partial sequences were assigned to the GH5-5 subfamily. Concerning the 23 different Basidiomycota AA2-sequence clusters obtained, 16 corresponded to MnP, 4 to GP and 3 to LiP. No VP sequence was detected. For each soil sample, the number of sequence clusters detected with the newly degenerate primers for families GH5 (subfamily 5), GH11 and AA2 was similar or higher to the number of sequence clusters obtained with the already published and widely used GH7 primers (S3 Table).

### MiSeq amplicon sequencing of multiple fungal lignocellulolytic gene family from soil cDNA samples

For the Illumina MiSeq sequencing, GH7, GH11 and AA2 PCR products obtained from one cDNA sample were pooled prior sequencing and a total of 12934 and 5761 assembled pair-reads (without “N”s) were obtained for the BS2010 and BB2010 soil samples, respectively. To ensure the highest quality of the datasets we removed assembled pair-reads containing primer mismatches or homopolymers and all suspected chimeric sequences to retain a total of 8910 (BS2010) and 2425 (BB2010) sequences for further analyses (Table 2). To determine at which percentage of identity sequences should be clustered, we first plotted for each gene family the number of sequence clusters against the percentage of identity used for clustering and then calculated the differences in the number of sequence clusters at n+1% identity versus the one at n% (S1 Fig.). The cutoff value was systematically set at the beginning of the plateau when the difference in the number of sequence clusters between two successive percentages of dissimilarity represented less than 6% of the number of clusters obtained at the cutoff value of 0% dissimilarity (S1 Fig.). As a result, sequences were clustered at a similarity cutoff value of 95% for the GH11 (S1-A Fig.) and GH7 sequences (S1-B Fig.) and of 93% for the AA2 ones (S1-C Fig.).

**Table 2:** Illumina MiSeq sequencing results obtained for Breuil Spruce (BS2010) and Breuil Beech (BB2010) forest soils.

	BS2010 GH7	BB2010 GH7	BS2010 GH11	BB2010 GH11	BS2010 AA2	BB2010 AA2
**Total no. of sequences**	1068	490	6060	1382	1782	553
**No. of clusters including singletons**	115	38	84	23	102	19
**No. of singletons (%)**	67 (58%)	26 (68%)	53 (63%)	11 (48%)	60 (59%)	10 (53%)
**No. of sequences used in subsamples with (without) singletons**	490 (465)	490 (465)	1382 (1372)	1382 (1372)	553 (542)	553 (542)
**No. of clusters in subsamples with (without) singletons**	83 (41)	38 (13)	40 (24)	23 (13)	49 (39)	19 (9)
**Shannon index with (without) singletons** 1	3.34 (3.01)	1.87 (1.58)	2.38 (2.27)	1.43 (1.38)	2.83 (2.77)	1.69 (1.59)
**S_chao1_ with (without) singletons** 1	251 (44)	103 (13)	82 (31)	41 (13)	82 (48)	64 (9)

Sequence statistics, diversity indexes and richness estimators calculated on subsamples generated from the initial sequence datasets (including singletons) or from datasets without the singletons.

All functional genes encode lignocellulolytic enzymes, i.e. fungal cellulases (GH7), fungal endo-β-1,4-xylanases (GH11), and Basidiomycota class-II peroxidases (AA2).

1 Shannon indexes and S_chao1_ estimators were calculated using datasets, which contained identical number of sequences for both forest soils.

Altogether, between 19 and 115 different sequence clusters were detected within the initial datasets (Table 2). Clusters containing a single sequence (i.e. so-called singletons) represented between 48 and 68% of these sequence clusters (Table 2). As 20% of these singletons contained at least one “stop codon” in the predicted ORF (data not shown), all unique sequences detected in only one soil sample were assumed to result from sequencing errors and removed from the initial datasets before further analyses. To control that removing singletons from the initial datasets did not modify their diversity patterns, we computed the Shannon indexes of each dataset including or not these singletons (Table 2). For each gene family, each dataset was first rarefied to the same sequence depth (i.e. the lowest sequence value between the two forest soils) before index calculation to eliminate the effect of sequencing effort. Similar Shannon indexes were observed for all datasets irrespective of the presence or absence of singletons (Table 2).

**Figure 2:**
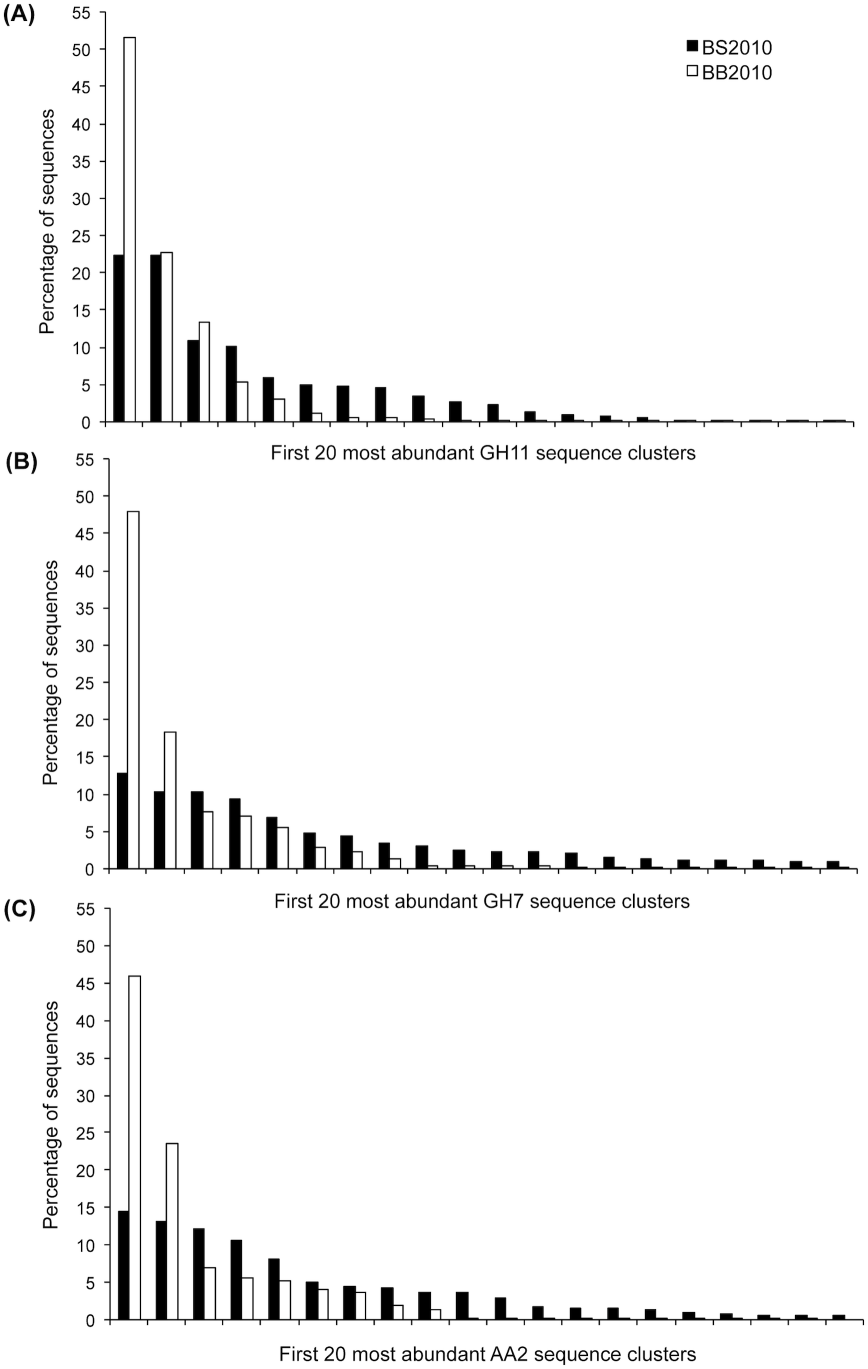
Distribution of the Illumina MiSeq sequences within the 20 first most abundant GH11 (A), GH7 (B) and AA2 (C) sequence clusters. Nucleotide sequences obtained from the two studied forest soils collected in 2010 under spruce (BS2010) and beech (BB2010) were clustered at 95 (GH11 and GH7) or 93% (AA2) identity threshold.

Higher gene diversity was systematically observed for the spruce forest stand. In the case of the AA2 gene family, the predicted number of sequence clusters (S_Chao1_ estimator) for the AA2 family was estimated at 48 for the spruce soil sample (BS2010) and only at 9 for the beech soil sample (BB2010). As illustrated in Fig. 2, ∼16 to 34% of the non-singleton sequence clusters contained 75% of the sequences. The percentage of shared sequence clusters between the two forest soils was low as it represented between 2 to 10% of the total number of clusters; which corresponded to only between 1 and 24% of the total number of sequences.

### Phylogenetic analysis of the environmental sequences

**Figure 3:**
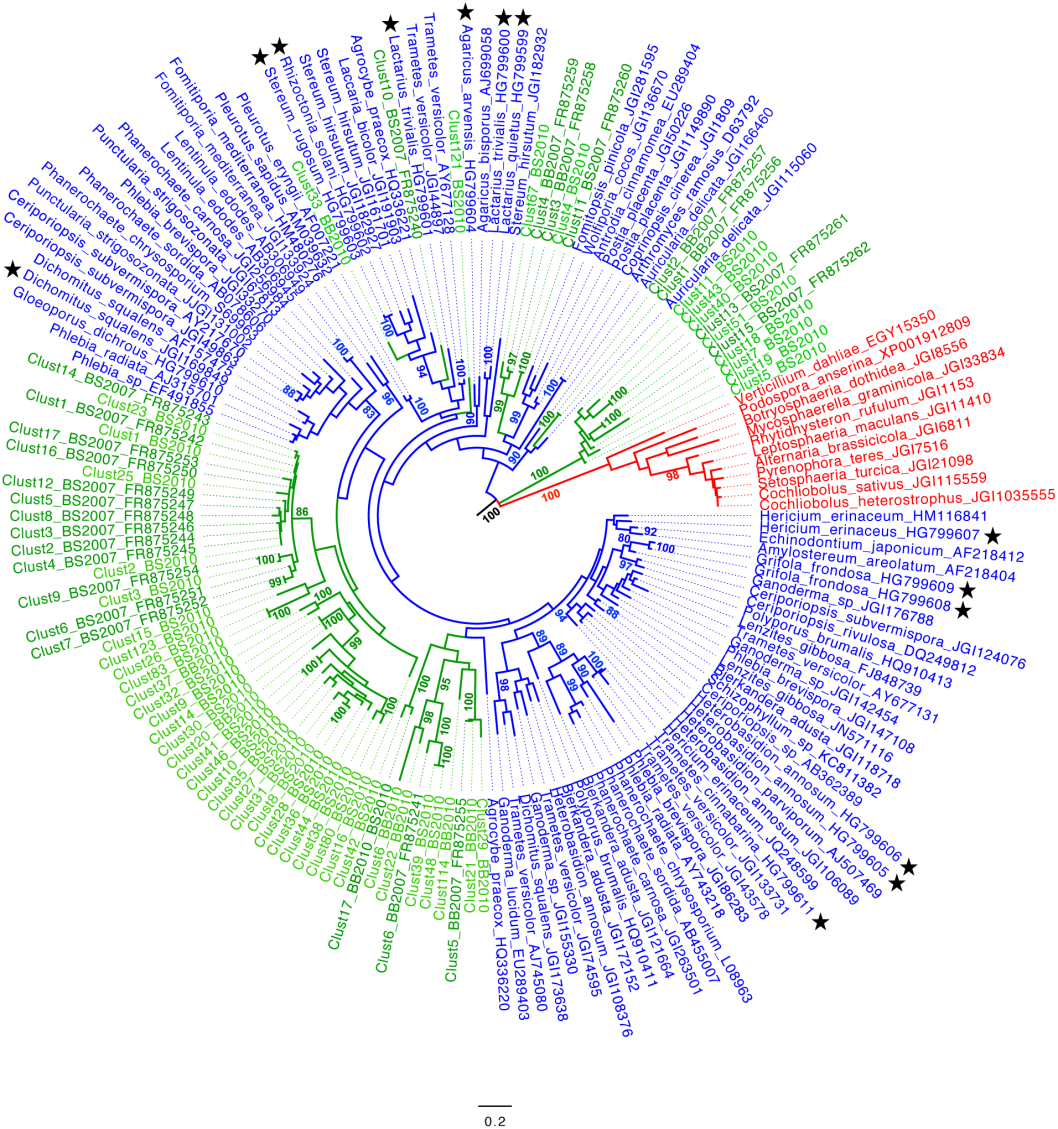
Phylogenetic positions of Basidiomycota class II peroxidase (AA2) amino-acid sequences. Basidiomycota AA2 amino-acid sequences were deduced from the nucleotide sequences amplified from beech (BB) or spruce (BS) soil cDNAs. The Maximum-likelihood phylogenetic tree includes all Sanger sequences amplified from the 2007 soil cDNA samples (BS2007 and BB2007) and all non-singleton sequence clusters detected by Illumina MiSeq sequencing of the 2010 soil cDNA samples (BS2010 and BB2010). Representative Ascomycota and Basidiomycota sequences are marked in red and blue, respectively, whereas the environmental sequences appear in green. Stars identify reference sequences obtained in the present study. Robustness of the tree topology was tested by bootstrap analysis (1000 replicates) and only bootstrap values ≥80 are given.

**Figure 4:**
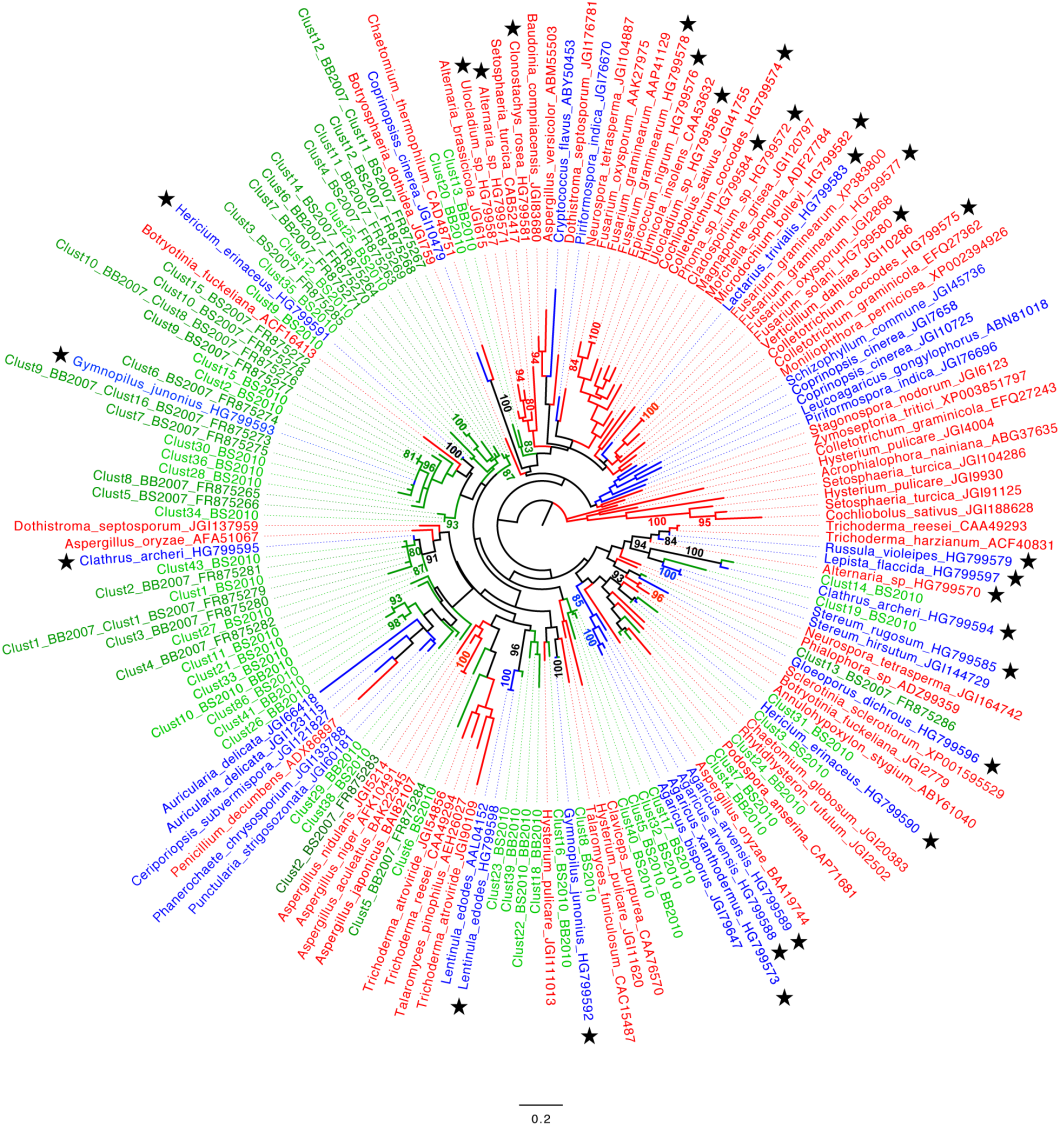
Phylogenetic positions of fungal endo-β-1,4-xylanase (GH11) amino-acid sequences. Fungal GH11 amino-acid sequences were deduced from the nucleotide sequences amplified from beech (BB) or spruce (BS) soil cDNAs. The Maximum-likelihood phylogenetic tree includes all Sanger sequences amplified from the 2007 soil cDNA samples (BS2007 and BB2007) and all non-singleton sequence clusters detected by Illumina MiSeq sequencing of the 2010 soil cDNA samples (BS2010 and BB2010). Representative Ascomycota and Basidiomycota sequences are marked in red and blue, respectively, whereas the environmental sequences appear in green. Stars identify reference sequences obtained in the present study. Robustness of the tree topology was tested by bootstrap analysis (1000 replicates) and only bootstrap values ≥80 are given.

Deduced amino-acid sequences from the soil cDNA sequences obtained by either Sanger or MiSeq sequencing were used for phylogenetic analyses. For the MiSeq sequences, the analysis was limited to the non-singleton sequence clusters of families GH7, GH11 and AA2. In addition to these environmental sequences, we also included in the alignments sequences obtained in the present study from fungal-extracted DNA as well as published fungal sequences representative of the diversity of each gene family. For both the AA2 (Fig. 3) family and GH5-5 (S2 Fig.) subfamily, a clear separation between Ascomycota and Basidiomycota sequences was observed in phylogenetic analyses, thus allowing confident assignation of anonymous environmental sequences to these taxa. This was clearly not the case for the GH11 (Fig. 4) and GH7 (S3 Fig.) gene families for which sequences from Ascomycota and Basidiomycota intermingled in the phylogenetic trees. Sequences, amplified using the newly designed GH5-5, GH11 and AA2 primers (from either fungal DNA or soil cDNA) were distributed over the entire corresponding gene trees. However, as manifest in the AA2 (Fig. 3) and GH11 (Fig. 4) phylogenetic gene trees, the environmental sequences tend to group together. Only few environmental sequences were found closely related to known reference ones. Few environmental sequences (i.e. 6 for the AA2, 5 for the GH5-5 and 3 for the GH7 family) detected in the soil samples collected in 2007 were also found in the soil samples collected in 2010.

## Discussion

Decomposition of plant organic matter is an enzymatically complex process largely mediated by consortia of fungal species that act simultaneously or successively [15], [55] and which need to be followed as they can be affected by several environmental factors [16], [56]–[57].

In the present study, we designed three pairs of degenerate primers to specifically amplify fungal lignocellulolytic genes (belonging to the GH5-5 subfamily, GH11 and AA2 families) involved in the hydrolysis of complex plant polymers (cellulose, hemicelluloses and lignins respectively) and used two of them (GH11 and AA2) along with the available GH7 primers [23] in a preliminary high-throughput MiSeq sequencing. These new primers were designed to generate fragments compatible with the Illumina MiSeq approach (i.e. PCR fragments smaller than 400 bp). As the GH5 family encompasses several catalytic activities, as opposed to Kellner et al. [18], we specifically targeted the fungal subfamily GH5-5 known to only encode endo-β-1,4-glucanases active on cellulose [34]–[35]. Moreover, the Basidiomycota-AA2 primers were designed to potentially amplify all class II peroxidases subfamilies (i.e. MnP, LiP, VP and GP) and not only the MnP one. The increasing number of fungal genomes and their taxonomic breadth [43] allowed us to improve the effectiveness and the specificity of the newly designed primers. Effectiveness of these degenerate primers was demonstrated by the relatively high correspondence (above 69%) between the known presence of these genes in 27 sequenced fungal genomes and their positive amplification from the DNA extracted from these species (Fig. 1). Effectiveness of these primers is also apparent from the broad distribution of the sequences, amplified either from fungal DNA or soil cDNA, across the different corresponding gene trees, which summarize the phylogenetic diversity of each gene family. Full primer universality is however impossible to achieve as experimentally demonstrated by e.g. Hadziavdic et al. [58] for the non-coding 18S rRNA gene widely used in metabarcoding of eukaryotic microbial communities. The situation is even worse for protein-coding gene families, which show better conservation at the amino-acid level than at the nucleotide one due to the degeneracy of the genetic code. Protein-coding gene families, such as those coding for lignocellulolytic enzymes also display complex evolutionary histories leading to the presence of highly variable numbers and sometimes unrelated homologous genes within and between fungal genomes [40], [59]–[60]. As a consequence the design of degenerate primers allowing the amplification of all gene copies from all species represents an unachievable aim. Based on the sequenced fungal genomes, we estimated that 33 to 100% of the gene copies were amplifiable with the GH5-5 designed primers and 33 to 50% for the GH11 ones. Concerning the AA2 family, if all subfamilies were amplified, our primers seemed to preferentially target LiP and MnP encoding genes (S2 Table). Specificity of the designed degenerate primers was confirmed as only the targeted genes were amplified from soil cDNA (results from either Sanger or Illumina MiSeq sequencing).

A majority of recent studies using degenerate primers targeted a single expressed functional gene family and sequenced amplicons using either the Sanger or the 454 pyrosequencing approach [26]–[27], [32]. Due to (i) the increasing length of the sequenced fragments (at present 2×300 bp), (ii) the very high output (25 million of reads per run) and (iii) the fixed length of the reads generated by the Illumina MiSeq platform, this NGS technology is replacing the pyrosequencing as the method of choice for low-cost and high-quality sequencing [61]. As such, the present study is the first one, which evaluates simultaneously the diversity of transcribed fungal genes encoding different enzymes active on plant cell wall polymers (cellulose, hemicelluloses and lignins) using the Illumina MiSeq technology. The matrix was soil RNA which contains a low proportion of mRNA, estimated at less than 10% by Urich et al. [62] and far less if we only consider eukaryotic mRNA [63]. Among these mRNA, fungal transcripts encoding specific categories of lignocellulolytic enzymes represent themselves a small proportion that has been estimated by systematic sequencing of forest soil eukaryotic cDNAs [64]. Among c.a. 16,000 cDNAs from the same spruce and beech soils used in the present study, only between two to seven transcripts corresponding to CAZy families GH5, GH7, GH11 and AA2 were identified [64]. Therefore, amplification of targeted sequences with degenerate primers combined with high-throughput sequencing certainly represents the most straightforward way to assess the diversity of specific functional gene categories in soils [33].

To analyze the diversity of any environmental sequence dataset obtained by metabarcoding, two parameters must be evaluated; (i) at which percentage of identity should be clustered the sequences and (ii) what is the biological significance of singletons and should they be taken into consideration? Regarding sequence clustering, it cannot be done at a fixed cutoff for all gene families as different genes evolve at different evolutionary rates [65]. Fungal lignocellulolytic gene families have complex evolutionary histories (characterized by multiple independent gene loss/acquisition events affecting homologous copies) leading to the presence of one or several, either highly similar or divergent, copies per genome [40], [59]–[60], [66]. As a consequence we empirically defined a different cutoff for each of the gene families as described in the result section. These cutoffs of 95% identity between DNA sequences for the GH7 & GH11 sequences and of 93% for the AA2 ones are somehow lower compared to those usually adopted in metabarcoding for non-translated rRNA sequences (usually ≥97%; [67]). This may reflect the coding nature of the corresponding sequences and the associated degeneracy of the genetic code. Interestingly, by clustering the GH7 sequences at a 95% identity threshold, we predicted a number of sequence clusters for the BS2010 spruce soil of 44 (Chao1 richness estimator, Table 2), a value similar to the 46±9 GH7 clusters per sample estimated by Baldrian et al. [33] for the humic horizon of another spruce forest soil.

Concerning the ecological significance of singletons, as about 20% of them contained at least one “stop codon” in the predicted ORF, we assumed that a majority of them may have arisen from sequencing errors. Moreover, as for other NGS datasets (e.g. [68]), Shannon diversity indices calculated for each functional gene family after rarefying the datasets from both forest soils to the same sequencing depth showed similar values independently of the presence or absence of these singletons (Table 2). We therefore opted for not taking into account these sequences. Furthermore, considering the typical distribution of fungal-taxa abundances in soils where only few taxa are highly abundant [67]–[69], for the low abundance taxa, even if they are highly active, their sequences will be likely often retrieved as singletons. The biological/technical significance of singletons is thus questionable and will always be affected by sequencing depth and errors [70].

As for the assignation of soil functional gene sequences to fungal taxa, it presently suffers from a lack of sequence information in public databases [22]–[23], [27], [32] despite recent efforts to sequence the genomes of fungal species representative of the diversity of this taxonomic group [43]. Most amplified environmental sequences indeed do not tightly cluster with sequences retrieved from public databases (GenBank, CAZy, JGI, Broad Institute databases) (Figs. 3–4 & S2–S3). Furthermore, only the AA2 and GH5-5 sequences originating from Ascomycota and Basidiomycota form well separated clades allowing unambiguous assignation of homologous environmental sequences to one of these two broad fungal groups (Figs. 3 & S2). Absence of tight association between environmental sequences and reference ones was particularly pronounced for the AA2 gene family (Fig. 3). One likely explanation is that AA2 peroxidases have essentially been studied in the context of wood degradation which has promoted the genome sequencing of many wood degrading saprotrophic species [37], [40] and not of unrelated soil saprotrophs. Indeed, as recently evaluated, the PeroxiBase and GenBank databases contained 311 entries of class II (AA2) peroxidases from wood-decay fungi versus only 11 from litter-decomposing species [27]. These figures plead for increasing the sequencing effort of reference sequences from soil inhabiting fungi, especially those belonging to the Basidiomycota.

Finally, the results we obtained for the spruce and beech forest soil samples are coherent with the fungal taxonomic survey performed by Buée et al. [69] on the same forest plots. Indeed, Buée et al. [69] demonstrated a higher fungal diversity in the spruce compared to the beech soils (983 versus 581 operational taxonomic units), and latter, Buée et al. [56] also showed that saprotrophic macromycete species were more abundant under spruce than under beech where ectomycorrhizal taxa dominated. In the present study, we also identified higher numbers of expressed gene sequence clusters for all three studied gene families in the spruce samples (Table 2) which indeed may reflect a predominance and greater abundance of saprotrophic species in the corresponding forest soils.

## Conclusions

In the present study, we designed primers targeting three functional fungal gene families encoding key enzymes involved in plant organic matter degradation (i.e. GH5-5, GH11 and AA2). We demonstrated their suitability for high-throughput sequencing using the Illumina MiSeq approach. We also evaluated two important parameters associated with the high-throughput sequencing (i) at which percentage of identity should be clustered coding nucleotide sequences and (ii) the biological significance of singletons. Such an approach constitutes a robust method, which allows a detailed characterization of the diversity of soil expressed fungal genes involved in plant organic matter degradation and may lead to the discovery of patterns in gene expression by soil fungal communities that may go unnoticed using other traditional approaches.

## Supporting Information

S1 Figure **Relation between the clustering threshold and the number of fungal endo-β-1,4-xylanase (GH11), cellulase (GH7) and Basidiomycota class II peroxidase (AA2) sequence clusters and of their “delta values”.** Evolution of the number of GH11 (A), GH7 (B) and AA2 (C) sequence clusters (N) expressed in soils (gray curve) and of their “delta values” (black curve) according to the percentage of dissimilarity used as cutoff for sequence clustering. “Delta values” represent the number of clusters at a cutoff of n% minus the values at n−1% (Δ = N_n%_–N_n−1%_).
(TIF)

S2 Figure **Phylogenetic positions of fungal endo-β-1,4-glucanase (GH5-5) amino-acid sequences.** Fungal GH5-5 amino-acid sequences were deduced from the nucleotide sequences amplified from beech (BB) or spruce (BS) soil cDNAs. The Maximum-likelihood phylogenetic tree include all Sanger sequences amplified from the 2007 soil cDNA samples (BS2007 and BB2007) and all non-singleton sequence clusters detected by Illumina MiSeq sequencing of the 2010 soil cDNA samples (BS2010 and BB2010). Representative Ascomycota and Basidiomycota sequences are marked in red and blue, respectively, whereas the environmental sequences appear in green. Stars identify reference sequences obtained in the present study. Robustness of the tree topology was tested by bootstrap analysis (1000 replicates) and only bootstrap values ≥80 are given.
(TIF)

S3 Figure **Phylogenetic positions of fungal cellulase (GH7) amino-acid sequences.** Fungal GH7 amino-acid sequences were deduced from the nucleotide sequences amplified from beech (BB) or spruce (BS) soil cDNAs. The Maximum-likelihood phylogenetic tree include all Sanger sequences amplified from the 2007 soil cDNA samples (BS2007 and BB2007) and all non-singleton sequence clusters detected by Illumina MiSeq sequencing of the 2010 soil cDNA samples (BS2010 and BB2010). Representative Ascomycota and Basidiomycota sequences are marked in red and blue, respectively, whereas the environmental sequences appear in green. Stars identify reference sequences obtained in the present study. Robustness of the tree topology was tested by bootstrap analysis (1000 replicates) and only bootstrap values ≥80 are given.
(TIF)

S1 Table **Reference sequences used for blastp search in NCBI and selected GenBank sequences for degenerate primer design.**
(DOCX)

S2 Table **Fungal species used to evaluate the degenerate primer efficiency and summary of sequencing results.**
(XLSX)

S3 Table **Analysis of the GH5-5, GH7, GH11 and AA2 sequences (Sanger sequencing) amplified from the 2007 forest soil cDNAs (Breuil Spruce (BS2007) and Breuil Beech (BB2007)).**
(DOCX)
