# Supplementary figures and images for: PCR Primers to Study the Diversity of Expressed Fungal Genes Encoding Lignocellulolytic Enzymes in Soils Using High-Throughput Sequencing
Source: PLoS One. 2014 Dec 29;9(12):e116264. doi: 10.1371/journal.pone.0116264 (PMC4278862; doi:10.1371/journal.pone.0116264)

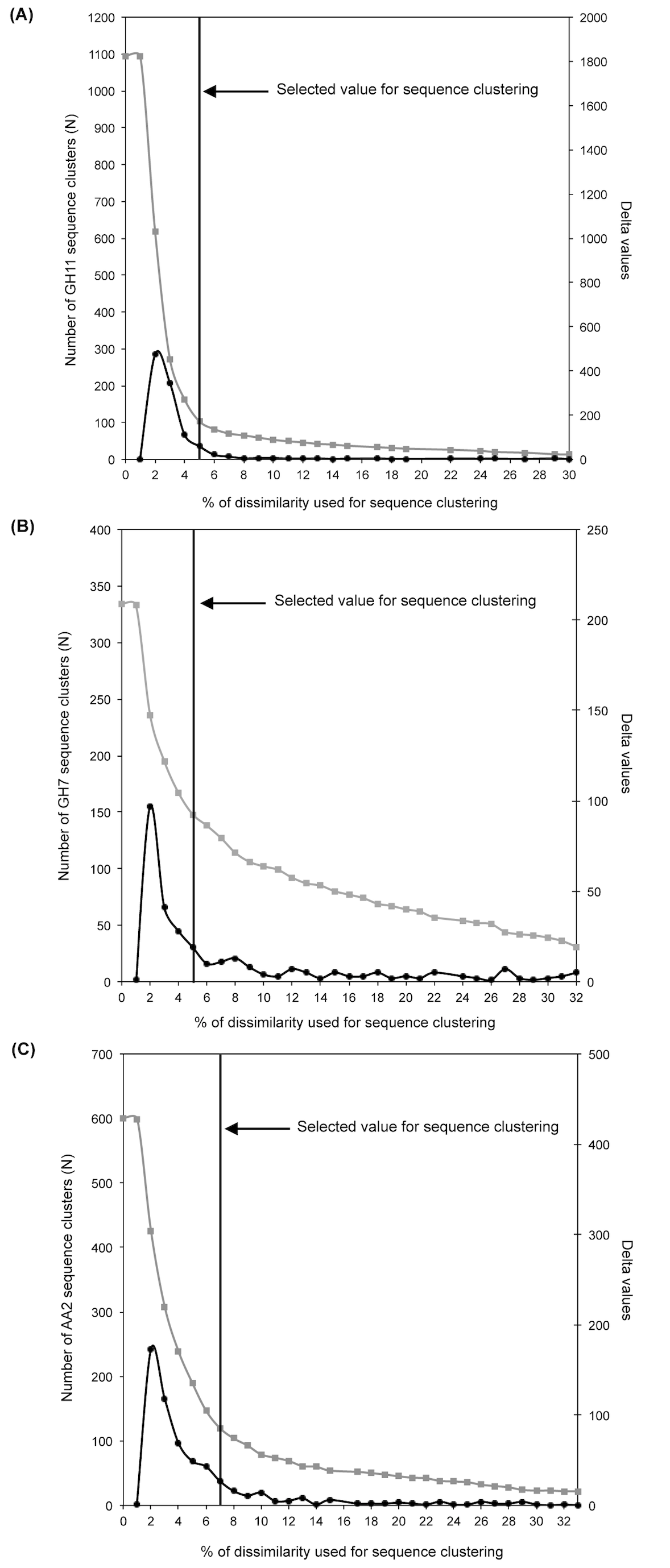

Supplement: S1 Figure — Relation between the clustering threshold and the number of fungal endo-β-1,4-xylanase (GH11), cellulase (GH7) and Basidiomycota class II peroxidase (AA2) sequence clusters and of their “delta values”. Evolution of the number of GH11 (A), GH7 (B) and AA2 (C) sequence clusters (N) expressed in soils (gray curve) and of their “delta values” (black curve) according to the percentage of dissimilarity used as cutoff for sequence clustering. “Delta values” represent the number of clusters at a cutoff of n% minus the values at n−1% (Δ = Nn%–Nn−1%). (TIF) [file pone.0116264.s001.tif]

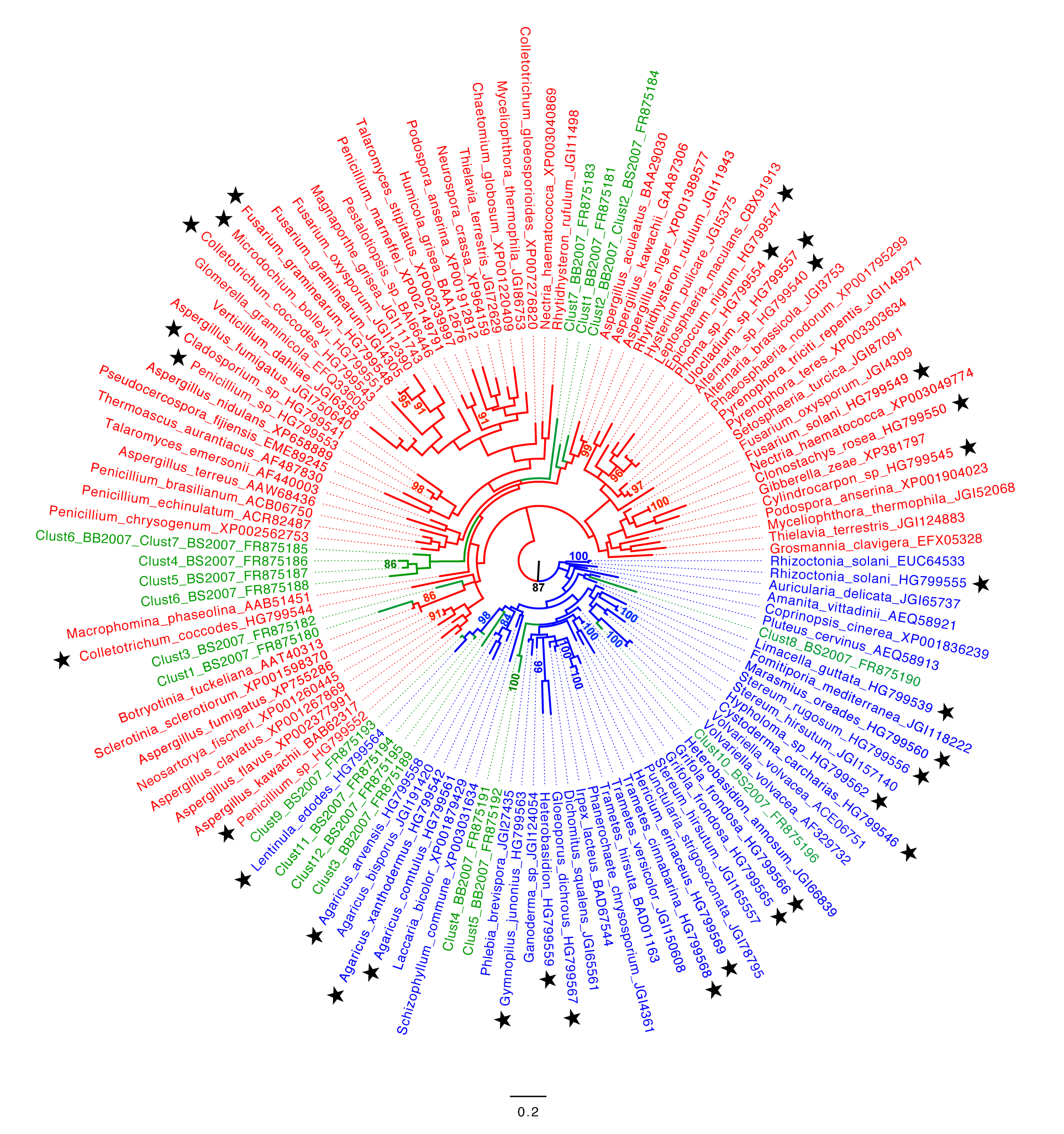

Supplement: S2 Figure — Phylogenetic positions of fungal endo-β-1,4-glucanase (GH5-5) amino-acid sequences. Fungal GH5-5 amino-acid sequences were deduced from the nucleotide sequences amplified from beech (BB) or spruce (BS) soil cDNAs. The Maximum-likelihood phylogenetic tree include all Sanger sequences amplified from the 2007 soil cDNA samples (BS2007 and BB2007) and all non-singleton sequence clusters detected by Illumina MiSeq sequencing of the 2010 soil cDNA samples (BS2010 and BB2010). Representative Ascomycota and Basidiomycota sequences are marked in red and blue, respectively, whereas the environmental sequences appear in green. Stars identify reference sequences obtained in the present study. Robustness of the tree topology was tested by bootstrap analysis (1000 replicates) and only bootstrap values ≥80 are given. (TIF) [file pone.0116264.s002.tif]

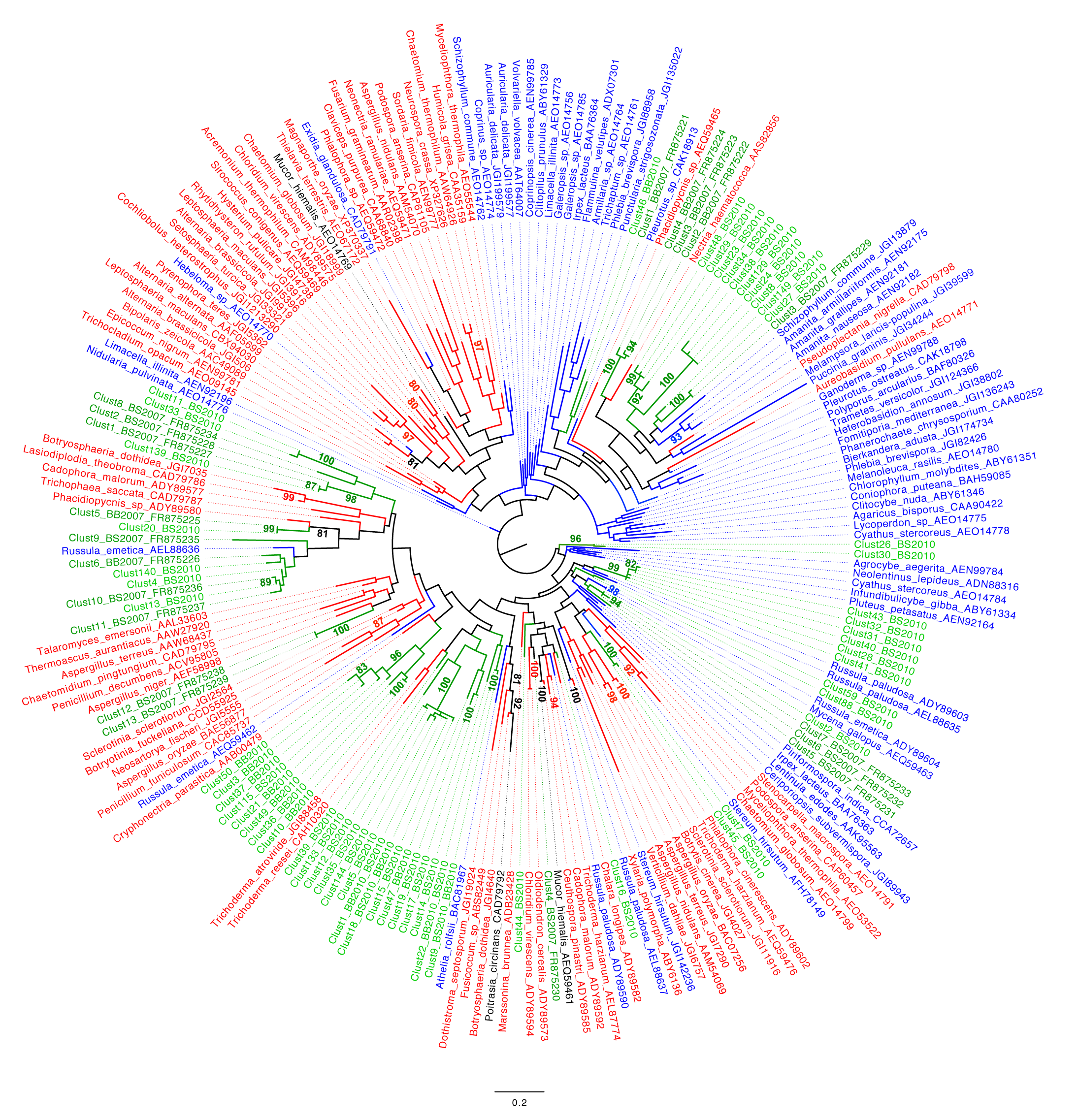

Supplement: S3 Figure — Phylogenetic positions of fungal cellulase (GH7) amino-acid sequences. Fungal GH7 amino-acid sequences were deduced from the nucleotide sequences amplified from beech (BB) or spruce (BS) soil cDNAs. The Maximum-likelihood phylogenetic tree include all Sanger sequences amplified from the 2007 soil cDNA samples (BS2007 and BB2007) and all non-singleton sequence clusters detected by Illumina MiSeq sequencing of the 2010 soil cDNA samples (BS2010 and BB2010). Representative Ascomycota and Basidiomycota sequences are marked in red and blue, respectively, whereas the environmental sequences appear in green. Stars identify reference sequences obtained in the present study. Robustness of the tree topology was tested by bootstrap analysis (1000 replicates) and only bootstrap values ≥80 are given. (TIF) [file pone.0116264.s003.tif]
