# Supplementary material for: PCR Primers to Study the Diversity of Expressed Fungal Genes Encoding Lignocellulolytic Enzymes in Soils Using High-Throughput Sequencing
Source: PLoS One. 2014 Dec 29;9(12):e116264. doi: 10.1371/journal.pone.0116264 (PMC4278862; doi:10.1371/journal.pone.0116264)
Supplement: S1 Table — Reference sequences used for blastp search in NCBI and selected GenBank sequences for degenerate primer design. (DOCX) [file pone.0116264.s004.docx]

**Table S1. Reference sequences used for blastp search in NCBI and selected GenBank sequences for degenerate primer design.**

| **Enzyme families/subfamilies** | **Accession numbers** | **Fungal groups** | **Fungal species** | **Conserved protein**  **regions 1** | **Conserved protein**  **regions 2** | **Conserved protein sequences selected for primer design** |
| --- | --- | --- | --- | --- | --- | --- |
|  |  |  |  |  |  | (Q/E)MHQYLD  &  WWAAGPW |
| **GH5-5** | BAD01163 ^1^ | Basidiomycota | *Trametes hirsuta* | QMHQYLD | WWAAGPW |  |
|  | XP_008039932 | Basidiomycota | *Trametes versicolor* | QMHQYLD | WWAAGPW |  |
|  | BAD67544 | Basidiomycota | *Irpex lacteus* | EMHQYLD | WWAAGPW |  |
|  | ETW82970 | Basidiomycota | *Heterobasidion irregulare* | EMHQYLD | WWAAGPW |  |
|  | XP_007368133 | Basidiomycota | *Dichomitus squalens* | QMHQYLD | WWAAGPW |  |
|  | KDR76291 | Basidiomycota | *Galerina marginata* | QMHQYLD | WWAAGPW |  |
|  | XP_007378214 | Basidiomycota | *Punctularia strigosozonata* | EMHQYLD | WWAAGQW |  |
|  | XP_007393231 | Basidiomycota | *Phanerochaete carnosa* | EMHQYLD | WWAAGPW |  |
|  | XP_007347849 | Basidiomycota | *Auricularia delicata* | EMHQYLD | WWAAGPW |  |
|  | XP_007300340 | Basidiomycota | *Stereum hirsutum* | EMHQYLD | WWAAGQW |  |
|  | KDQ10377 | Basidiomycota | *Botryobasidium botryosum* | EMHQYLD | WWAAGPW |  |
|  | CDJ79817 | Basidiomycota | *Leucoagaricus gongylophorus* | EMHQYLD | WWAAGPW |  |
|  | XP_007263372 | Basidiomycota | *Fomitiporia mediterranea* | EMHQYLD | WWAAGPW |  |
|  | KEP51038 | Basidiomycota | *Rhizoctonia solani* | EMHQYLD | WWAAGPW |  |
|  | XP_001879429 | Basidiomycota | *Laccaria bicolor* | EMHQYLD | WWAAGPW |  |
|  | XP_006459448 | Basidiomycota | *Agaricus bisporus* | QMHQYLD | WWSAGPW |  |
|  | CCX05395 | Ascomycota | *Pyronema omphalodes* | EMHQYLD | WWAAGPW |  |
|  | XP_003031634 | Basidiomycota | *Schizophyllum commune* | EMHQYLD | WWAAGPW |  |
|  | XP_007304563 | Basidiomycota | *Stereum hirsutum* | EMHQYLD | WWAAGPW |  |
|  | XP_007851566 | Basidiomycota | *Moniliophthora roreri* | EMHQYLD | WWAAGPW |  |
|  | KDQ27083 | Basidiomycota | *Pleurotus ostreatus* | EMHQYLD | WWAAGPW |  |
|  | XP_002840457 | Ascomycota | *Tuber melanosporum* | EMHQYLD | WWAAGPW |  |
|  | XP_001836239 | Basidiomycota | *Coprinopsis cinerea* | QMHQYLD | WWAAGPW |  |
|  | XP_003654855 | Ascomycota | *Thielavia terrestris* | EMHQYLD | WWGAGPW |  |
|  | XP_007581661 | Ascomycota | *Neofusicoccum parvum* | QMHQYLD | WWAAGPW |  |
|  | EGX49988 | Ascomycota | *Arthrobotrys oligospora* | EMHQYLD | WWAAGPW |  |
|  | XP_001934618 | Ascomycota | *Pyrenophora tritici-repentis* | QMHQYLD | WWAAGPW |  |
|  | EFX05328 | Ascomycota | *Grosmannia clavigera* | EMHQYLD | WWGAGPW |  |
|  | XP_003049774 | Ascomycota | *Nectria haematococca* | EMHQYLD | WWAAGPW |  |
|  | XP_003663700 | Ascomycota | *Myceliophthora thermophila* | EMHQYLD | WWAAGPW |  |
|  | XP_007290718 | Ascomycota | *Marssonina brunnea* | EMHQYLD | WWAGGPW |  |
|  | AAL33630 | Ascomycota | *Rasamsonia emersonii* | EMHQYLD | WWAAGPW |  |
|  | XP_381797 | Ascomycota | *Fusarium graminearum* | QMHQYLD | WWAAGPW |  |
|  | EPS30243 | Ascomycota | *Penicillium oxalicum* | EMHQYLD | WWAAGPW |  |
|  | EFQ31119 | Ascomycota | *Colletotrichum graminicola* | EMHQYLD | WWAAGPW |  |
|  | AHY00945 | Ascomycota | *Aspergillus niger* | EMHQYLD | WWAAGPW |  |
|  | EME41652 | Ascomycota | *Dothistroma septosporum* | QMHQYLD | WWAAGPW |  |
|  | ESZ99399 | Ascomycota | *Sclerotinia borealis* | EFHQYLD | WWAAGPW |  |
|  | AFY98622 | Ascomycota | *Bispora antennata* | EMHQYLD | WWGGGPW |  |
|  | AAT40313 | Ascomycota | *Botrytis cinerea* | EFHQYLD | WWGAGPW |  |
|  | XP_001904023 | Ascomycota | *Podospora anserina* | QMHQYLD | WWGGGPW |  |
|  | XP_002149791 | Ascomycota | *Talaromyces marneffei* | EMHQYLD | FWAAGPW |  |
|  | EKG11044 | Ascomycota | *Macrophomina phaseolina* | QMHQYLD | WWAAGPW |  |
|  | KEQ63578 | Ascomycota | *Aureobasidium melanogenum* | QMHQYLD | WWAAGPW |  |
|  | AEQ58918 | Basidiomycota | *Amanita prairiicola* | QMHQYLD | WWAAGPW |  |
|  | AEQ58912 | Basidiomycota | *Limacella illinita* | QMHQYLD | WWAAGPW |  |
|  | AEQ58913 | Basidiomycota | *Pluteus cervinus* | EMHQYLD | WWAAGPW |  |
|  | AEQ58915 | Basidiomycota | *Pluteus petasatus* | EMHQYLD | WWAAGPW |  |
|  | AEQ58914 | Basidiomycota | *Volvariella volvacea* | EMHQYLD | WWAAGPW |  |
|  | XP_007795848 | Ascomycota | *Eutypa lata* | DIHEYLD | WWAAGPW |  |
|  | XP_006692513 | Ascomycota | *Chaetomium thermophilum* | EMHQYLD | WWAAGPW |  |
|  | KDQ30980 | Basidiomycota | *Pleurotus ostreatus* | DVHKYLD | VWSAGSF |  |
|  | XP_008084003 | Ascomycota | *Glarea lozoyensis* | DIHEYLD | AWAAGPF |  |
|  |  |  |  |  |  | GKGWNPG  &  QYWS(I/V)RQ |
| **GH11** | AAL04152 ^1^ | Basidiomycota | *Lentinula edodes* | GKGWNPG | QYWSIRQ |  |
|  | XP_007397319 | Basidiomycota | *Phanerochaete carnosa* | GKGWNPG | QYWSIRS |  |
|  | ABZ88798 | Basidiomycota | *Phanerochaete chrysosporium* | GKGWNPG | QYWSIRS |  |
|  | ADZ99359 | Ascomycota | *Phialophora* sp. | GKGWNPG | QYWSVRR |  |
|  | XP_007379856 | Basidiomycota | *Punctularia strigosozonata* | GKGWNPG | QYWSIRS |  |
|  | XP_007300161 | Basidiomycota | *Stereum hirsutum* | GKGWNPG | QYWSVRQ |  |
|  | XP_007343831 | Basidiomycota | *Auricularia delicata* | GKGWNPG | QYFSIRQ |  |
|  | XP_001258363 | Ascomycota | *Neosartorya fischeri* | GKGWNPG | QYWSVRT |  |
|  | EME39847 | Ascomycota | *Dothistroma septosporum* | GKGWNPG | QYWSIRT |  |
|  | CAC15487 | Ascomycota | *Talaromyces funiculosus* | GKGWNPG | QYWSVRT |  |
|  | XP_001389848 | Ascomycota | *Aspergillus niger* | GKGWNPG | QYWSVRT |  |
|  | XP_008078781 | Ascomycota | *Glarea lozoyensis* | GKGWNPG | QYWSVRQ |  |
|  | BAE71133 | Ascomycota | *Penicillium citrinum* | GKGWNPG | QYWSVRQ |  |
|  | EMD39588 | Basidiomycota | *Ceriporiopsis subvermispora* | GKGWNPG | QYWSIRS |  |
|  | EFQ30380 | Ascomycota | *Colletotrichum graminicola* | GKGWNPG | QYWSVRQ |  |
|  | EMF09157 | Ascomycota | *Sphaerulina musiva* | GKGWNPG | QYWSIRT |  |
|  | ADF27784 | Ascomycota | *Morchella spongiola* | GKGWSPG | QYWSVRK |  |
|  | CCA76116 | Basidiomycota | *Piriformospora indica* | GKGWNPG | QFWSVRQ |  |
|  | BAO51921 | Ascomycota | *Talaromyces cellulolyticus* | GKGWNPG | QYWSVRT |  |
|  | KDR71791 | Basidiomycota | *Galerina marginata* | GKGWNPG | QYWSVRQ |  |
|  | EJT75607 | Ascomycota | *Gaeumannomyces graminis* | GKGWNPG | QYWAIRT |  |
|  | ETI21992 | Ascomycota | *Cladophialophora carrionii* | GKGWNPG | QYWSIRT |  |
|  | XP_003050975 | Ascomycota | *Nectria haematococca* | GKGWNPG | QYWSVRR |  |
|  | EGU83183 | Ascomycota | *Fusarium oxysporum* | GKGWMPG | QYWSVRR |  |
|  | AAP83925 | Ascomycota | *Trichoderma viride* | GKGWNPG | QYWSVRR |  |
|  | XP_008028976 | Ascomycota | *Setosphaeria turcica* | GKGWNPG | QFWSVRT |  |
|  | 164562254 | Basidiomycota | *Pleurotus ostreatus* | GKGWNPG | QYWSVRT |  |
|  | ESZ90340 | Ascomycota | *Sclerotinia borealis* | GKGWAVG | QYWSVRT |  |
|  | ABE02800 | Ascomycota | *Verticillium dahliae* | GKGWNPG | QYWSVRT |  |
|  | XP_001910545 | Ascomycota | *Podospora anserina* | GKGWNPG | QFWSVRR |  |
|  | XP_003662402 | Ascomycota | *Myceliophthora thermophila* | GKGWNPG | QFWSVRT |  |
|  | ABG33753 | Ascomycota | *Alternaria* sp. | GKGWNPG | QYWSVRT |  |
|  | XP_007912948 | Ascomycota | *Togninia minima* | GKGWNPG | QYWSIRQ |  |
|  | XP_003837356 | Ascomycota | *Leptosphaeria maculans* | GKGWNPG | QYWSVRQ |  |
|  | XP_007292301 | Ascomycota | *Marssonina brunnea* | GKGYKPG | QYWSVRR |  |
|  | KEQ91156 | Ascomycota | *Aureobasidium subglaciale* | GKGWNPG | QYWSVRQ |  |
|  | AHC72381 | Ascomycota | *Humicola insolens* | GKGWNPG | QYWSVRR |  |
|  | AAZ03776 | Ascomycota | *Botrytis cinerea* | GKGWAVG | QYWSVRT |  |
|  | XP_001941158 | Ascomycota | *Pyrenophora tritici-repentis* | GKGWNPG | QYWSVRT |  |
|  | XP_006690651 | Ascomycota | *Chaetomium thermophilum* | GKGWNPG | QYWSVRR |  |
|  | EHK25898 | Ascomycota | *Trichoderma virens* | GKGWNPG | QYWSVRR |  |
|  | GAD94166 | Ascomycota | *Byssochlamys spectabilis* | GKGWSTG | QYWSVRQ |  |
|  | ABG37634 | Ascomycota | *Acrophialophora nainiana* | GKGWNPG | QYWSVRQ |  |
|  | CAA76570 | Ascomycota | *Claviceps purpurea* | GRGWNPG | QYWSIRR |  |
|  | EUN22843 | Ascomycota | *Bipolaris victoriae* | GKGRNPG | QYWSVRQ |  |
|  | XP_007857498 | Ascomycota | *Moniliophthora roreri* | GKGWNPG | QFWSVRN |  |
|  | XP_003715734 | Ascomycota | *Magnaporthe oryzae* | GKGWNPG | QFWSVRR |  |
|  |  |  |  |  |  | GGGADGS  &  PFDSTP |
| **AA2** | AAA34049 ^1^ | Basidiomycota | *Trametes versicolor* | GGGADGS | PFDSTP |  |
|  | XP_008043740 | Basidiomycota | *Trametes versicolor* | GGGADGS | PFDSTP |  |
|  | XP_008043625 | Basidiomycota | *Trametes versicolor* | GGGADGS | PFDSTP |  |
|  | AEJ37998 | Basidiomycota | *Polyporus brumalis* | GGGADGS | PFDSTP |  |
|  | AEX01147 | Basidiomycota | *Lenzites gibbosa* | GGGADGS | PFDSTP |  |
|  | BAE79812 | Basidiomycota | *Spongipellis* sp. | GGGADGS | PFDSTP |  |
|  | 1906181A | Basidiomycota | *Bjerkandera adusta* | GGGADGS | PFDSTP |  |
|  | ADK26471 | Basidiomycota | *Hericium erinaceus* | GGGADGS | PFDSTP |  |
|  | BAG85350 | Basidiomycota | *Phanerochaete sordida* | GGGADGS | PFDSTP |  |
|  | XP_007365204 | Basidiomycota | *Dichomitus squalens* | GGGADGS | PFDSTP |  |
|  | AGO86670 | Basidiomycota | *Irpex lacteus* | GGGADGS | PFDSTP |  |
|  | BAG49629 | Basidiomycota | *Ceriporiopsis* sp. | GGGADGS | PFDSTP |  |
|  | ETW80422 | Basidiomycota | *Heterobasidion irregulare* | GGGADGS | PFDSTP |  |
|  | XP_007266673 | Basidiomycota | *Fomitiporia mediterranea* | GGGADGS | PFDSTP |  |
|  | ACM47219 | Basidiomycota | *Pleurotus ostreatus* | GGGADGS | PFDSTP |  |
|  | ETW82129 | Basidiomycota | *Heterobasidion irregulare* | SGGADGS | PFDSTP |  |
|  | XP_007845338 | Basidiomycota | *Moniliophthora roreri* | GGGADGS | PFDSTP |  |
|  | AFR44747 | Basidiomycota | *Volvariella volvacea* | GGGADGS | PFDSTP |  |
|  | AFK91531 | Basidiomycota | *Cerrena unicolor* | GGGADGS | PFDSTP |  |
|  | ADW41626 | Basidiomycota | *Agrocybe praecox* | GGGADGS | PFDSTP |  |
|  | ADK60913 | Basidiomycota | *Trametes cinnabarina* | GGGADGS | PFDSTP |  |
|  | ADK60911 | Basidiomycota | *Trametes cinnabarina* | GGGADGS | PFDSTP |  |
|  | XP_006460927 | Basidiomycota | *Agaricus bisporus* | GGGADGS | PFDSTP |  |
|  | XP_007309309 | Basidiomycota | *Stereum hirsutum* | GGGADGS | PFDSTP |  |
|  | ACA48488 | Basidiomycota | *Ganoderma lucidum* | GGGADGS | PFDSTP |  |
|  | KDR70719 | Basidiomycota | *Galerina marginata* | GGGADGS | PFDTSP |  |
|  | BAE46585 | Basidiomycota | *Trametopsis cervina* | GGGADGS | PFDSTP |  |
|  | ABT17196 | Basidiomycota | *Phanerochaete chrysosporium* | GGGADGS | PFDSTP |  |
|  | XP_007353069 | Basidiomycota | *Auricularia delicata* | GGGADGS | PFDSTP |  |
|  | AAW59419 | Basidiomycota | *Phlebia radiata* | GGGADGS | PFDSTP |  |
|  | XP_007382780 | Basidiomycota | *Punctularia strigosozonata* | GGGADGS | PFDSTP |  |

^1^ CAZy reference sequences used for blastp search
