# Supplementary material for: PCR Primers to Study the Diversity of Expressed Fungal Genes Encoding Lignocellulolytic Enzymes in Soils Using High-Throughput Sequencing
Source: PLoS One. 2014 Dec 29;9(12):e116264. doi: 10.1371/journal.pone.0116264 (PMC4278862; doi:10.1371/journal.pone.0116264)
Supplement: S3 Table — Analysis of the GH5-5, GH7, GH11 and AA2 sequences (Sanger sequencing) amplified from the 2007 forest soil cDNAs (Breuil Spruce (BS2007) and Breuil Beech (BB2007)). (DOCX) [file pone.0116264.s006.docx]

**Table S3. Analysis of the GH5-5, GH7, GH11 and AA2 sequences (Sanger sequencing) amplified from the 2007 forest soil cDNAs (Breuil Spruce (BS2007) and Breuil Beech (BB2007)).**

|  | **BS2007**  **GH5-5** | **BB2007**  **GH5-5** | **BS2007**  **GH7** | **BB2007**  **GH7** | **BS2007**  **GH11** | **BB2007**  **GH11** | **BS2007**  **AA2** | **BB2007**  **AA2** |
| --- | --- | --- | --- | --- | --- | --- | --- | --- |
| **No. of sequenced clones** | 48 | 48 | 48 | 48 | 48 | 48 | 48 | 48 |
| **No. of bad quality sequences** | 9 | 14 | 14 | 5 | 11 | 4 | 7 | 11 |
| **No. of chimeric sequences** | 0 | 0 | 0 | 0 | 0 | 0 | 0 | 22 |
| **No. of fungal sequences** | 39 | 34 | 34 | 43 | 37 | 44 | 41 | 15 |
| **No. of cluster (singletons)^1^** | 12 (6) | 7 (3) | 13 (4) | 6 (2) | 16 (8) | 12 (6) | 17 (11) | 6 (4) |

^1^ nucleotide sequences were clustered at 100% identity.
